# Impact of the COVID-19 pandemic on the Thai population: Delineating the effects of the pandemic and policy measures

**DOI:** 10.1017/ash.2023.523

**Published:** 2023-12-18

**Authors:** Nongyao Kasatpibal, Nongkran Viseskul, Akarapong Untong, Kwaunpanomporn Thummathai, Kampong Kamnon, Srisakul Sangkampang, Rusila Tokilay, Srisuda Assawapalanggool, Anucha Apisarnthanarak

**Affiliations:** 1 Division of Nursing Science, Faculty of Nursing, Chiang Mai University, Chiang Mai, Thailand; 2 Epidemiology Research Center of Infectious Disease (ERCID), Chiang Mai University, Chiang Mai, Thailand; 3 School of Tourism Development, Maejo University, Chiang Mai, Thailand; 4 Rajavithi Hospital, Bangkok, Thailand; 5 Lumthamenchai Hospital, Nakhon Ratchasima, Thailand; 6 Yala Provincial Public Health Office, Yala, Thailand; 7 Infection Control Section, Mae Sot Hospital, Thailand; 8 Division of Infectious Diseases, Thammasat University Hospital, Pratumthani, Thailand

## Abstract

**Objectives:**

This study aimed to determine the impacts of the COVID-19 pandemic and policy measures and delineate the impact of each on a cohort of Thai citizens.

**Methods:**

A cross-sectional study was conducted among 2,500 Thai people from October 2020 to January 2021. A questionnaire collecting demographic information and other data was sent to eligible subjects.

**Results:**

Overall, 51.6% and 49.5% of participants felt the impacts of COVID-19 and policy measures at the highest level, respectively. The study demonstrated that the weighted effect of the impact of the COVID-19 outbreak was statistically (*p* < .001) greater than that of policy measures on family (0.664 vs 0.618), education (0.562 vs 0.557), and the economy (0.643 vs 0.572). The weighted effect of the impact of policy measures was statistically (*p* < .001) greater than that of the COVID-19 pandemic on people’s daily activities (0.675 vs 0.651), cultural/traditional or community way of life (0.769 vs 0.736), access to healthcare services and infection prevention supplies (0.410 vs 0.390), and mental health (0.625 vs 0.584).

**Conclusions:**

About half of the participants had a high level of impact from both the COVID-19 pandemic and policy measures. The results of this study suggest that policy measures need to be judged with caution, and the government should provide more comprehensive support to reduce the impact on people’s lives.

## Introduction

The COVID-19 pandemic and policy measures such as lockdown, social distancing, and working or studying at home have had prolonged effects on people’s daily activities,^1,2^ families,^1^ culture/traditions or ways of life,^3^ education,^4^ access to healthcare services and infection prevention supplies,^5^ the economy,^1^ and mental health.^1,3^ A study on the social and cultural impacts of COVID-19 found that the pandemic caused a reduction in daily activities, certain religious activities, and traditional and cultural activities. It also kept friends and family members at a distance, resulting in a lack of social contact.^2^ In addition, patients, people under quarantine, caregivers, family, friends, and communities were exposed to social stigma due to lack of knowledge about the disease, resulting in paranoia and anxiety in society, social isolation, and a decrease in the solidarity and unity of people in society.^6,7^ A pilot study in Thailand demonstrated that the effects of the COVID-19 pandemic and government policies included a decrease in national travel (63.5%), eating out (63.0%), participation in religious, traditional and cultural activities (61.5%), patronage of beauty salons (61.0%), and shopping at the mall (58.0%).^8^

A study in Zambia found that the spread of COVID-19 affected the education of students at the high school level. The teachers and students lacked support for online learning. This resulted in a reduction in learning interactions between students and teachers. It also affected the students’ entrance examination scores to higher education.^9^ These findings were very similar to the findings of a pilot study in Thailand. Some students reported a delay in graduation (61.0%), did not have devices and accessories needed for online learning (43.0%), had no support for solving problems during online learning (43.0%), had difficulty accessing or had no internet access for online learning (38.5%), and lacked the skills for online learning (38.0%).^8^

The COVID-19 pandemic disrupted the global economy. It resulted in the economic slowdown of countries around the world, including Europe, the Americas, and Asia^10^ resulting in a decrease of gross domestic product and economic growth.^11^ It also slowed down the manufacturing of essential goods, disrupted supply chains, led to losses in national and international business, and resulted in poor cash flow for global markets.^2^ In addition, a report from the US revealed that COVID-19 caused a financial crisis among those with low incomes, with 43.0% of US adults losing their jobs or having their wages cut. As a result, 53.0% of US adults did not have enough funds to cover expenses in the first month after a job loss and only 23.0% expected that they had enough funds to get through a three-month period.^12^ Economic experts predicted that the impact could cause 420–580 million people worldwide to enter into poverty.^11^ A study of people in urban slums in Thailand found that 18.9% and 18.0% of working-aged participants were laid off or had reduced working time and income, respectively. Street food vendors could not earn income (18.2%), while those who were self-employed had reduced or no income (18.4%). A majority (60.2%) of the population had vastly decreased income and nearly a third of the population (31.2%) lost about half their income. Less than 10.0% felt little or no economic impact due to earning a fixed salary.^13^

Previous studies have demonstrated that the major impact of the COVID-19 pandemic has been psychological leading to increased fear, anxiety, stress, and depression leading to suicide.^14–19^ A study in China found that the frequency of social media exposure increased the risk of anxiety (OR = 1.72, 95% CI = 1.31–2.26) and both depression and anxiety (OR = 1.91, 95% CI = 1.52–2.41).^20^ A pilot study in Thailand found that the participants felt stress after being laid off (63.5%), stress from the fear of being unemployed (62.5%), anxiety about finding a new job (62.0%), anxiety about future layoffs (61.0%), and depression from social isolation (39.0%).^8^

COVID-19 and subsequent government policies have affected various aspects of people’s lives. However, few studies have compared the contribution of each. This study aimed to ascertain the impacts of the COVID-19 pandemic and resulting policy measures.

## Methods

### Study design and participants

Between October 2020 and January 2021, a cross-sectional study was conducted among 2,500 people living in 5 provinces in Thailand using a stratified sampling method. Participants who lived in Bangkok, Chonburi, Chiang Mai, Nakhon Ratchasima, and Yala provinces were selected because they experienced a notable surge in COVID-19 cases in each region during the study timeframe. All participants were at least 18 years of age, able to communicate in the Thai language, and were willing to cooperate with this study. Persons who were critically ill during the study period or who could not provide information for this study were excluded.

### Ethical considerations

The Research Ethics Committee at the Faculty of Nursing, Chiang Mai University (reference no. 105-2020) approved this study. Before signing an informed consent form, participants were made aware of the study’s objectives, procedures, and benefits. Data collection began only after participants had given their consent. Each participant’s identity was kept confidential.

### Instrument

The researchers developed a questionnaire as the research instrument, which underwent a thorough review to establish a clear theoretical framework for each impact domain. Two research consultants provided feedback, leading to reorganization of impact classification into 7 aspects. Following this, six experts assessed construct validity, ensuring face validity through a Zoom meeting where wording in some questions was revised. Pilot testing with 15 samples gathered feedback on question clarity, relevance, and appropriateness, leading to further refinement based on input from experts and the pilot testing.

The content validity index of the impact of the COVID-19 questionnaire was 1.00 and the reliabilities of the impact of the COVID-19 questionnaire and the depression anxiety stress questionnaire (DASS-21) translated into the Thai language DASS-21]^21^ were 0.84 and 0.83, respectively.

The questionnaire consists of three parts. Part 1: demographic information that included the province, age, gender, occupation, education level, religion, income, and level of compensation from the government. Part 2: the impact of the COVID-19 pandemic (the consequences of the spread of SARS-CoV-2) and policy measures (the consequences of the actions taken by governments, ministry of public health, and other organizations to mitigate the spread of COVID-19) questionnaire with a total of 48 items on a 4-point rating scale with responses of none (1 point), low (2 points), moderate (3 points), and high (4 points). The impact of the COVID-19 pandemic and policy measures was classified into 7 aspects including people’s daily activities, family, cultural/traditional or community ways of life, education, infection prevention and access to healthcare, economy, and mental health. The total impact score was further divided into 3 levels: low, moderate, and high. Part 3: the depression anxiety stress questionnaire asks the individual to indicate the presence of a symptom over the previous week. This questionnaire had a total of 21 items on a 4-point rating scale ranging from 0 (did not apply to me at all) to 3 (applied to me very much or most of the time) and was designed to measure the severity of a range of symptoms common to depression, anxiety, and stress. Responses were classified into 5 levels including normal, mild, moderate, severe, and extremely severe.

### Data collection

Village health volunteers distributed the questionnaire to 500 participants living in each of 5 provinces for a total of 2,500 participants. The response rate was 100%.

### Data analysis

Data were analyzed using R version 3.5.1. Frequency and percentage, mean and standard deviation, and median and range were calculated for demographic data, the impact of the COVID-19 pandemic, and policy measures data as appropriate. Factor loadings in confirmatory factor analysis were performed and *T*-statistics were used to test the overall weighted mean difference between the impact of the COVID-19 outbreak and policy measures and the differences in each province. The level of signiﬁcance was set at *p* < .05.

## Results

### Demographics

Most participants were female (71.6%), laborers (34.6%), and had a mean age of 43.4 ± 14.4 years. Most participants identified as Buddhist (78.0%). A majority of participants held a primary- or secondary-level education (35.2% and 34.0%, respectively). Most of them had mid-level incomes (83.7%). Most participants received compensation from the government (59.0%) (Table 1).

**Table 1.** Participant demographics (*n* = 2,500)

Characteristics	*n*	%
Sex		
Female	1,791	71.6
Male	709	28.4
Occupation		
Laborer	866	34.6
Farmer	444	17.8
Merchant	278	11.1
Company employee	220	8.8
Government officer	199	8.0
Student	121	4.8
Retired civil servant	83	3.3
Government employee	36	1.4
State enterprise employee	15	0.6
Unemployed	238	9.6
Age (years)		
≤30	649	26.0
31–40	421	16.8
41–50	527	21.0
51–60	579	23.2
>60	324	13.0
Mean = 43.4, SD = 14.4, Median = 44.5, Range = 18–70		
Religion		
Buddhist	1,951	78.0
Muslim	521	20.8
Christian	28	1.2
Highest education level		
Primary school	877	35.2
Secondary school	850	34.0
Diploma degree	231	9.2
Bachelor’s degree	501	20.0
Master’s degree or higher	41	1.6
Income		
High	23	0.9
Middle	2,092	83.7
Low	330	13.2
None	55	2.2
Compensation from the government		
Received	1,496	59.0
Not received	962	38.5
Unknown	62	2.5

### Level of impact on COVID-19 from the pandemic and policy measures

Overall, participants reported a high level of impact from the COVID-19 pandemic and the resulting policy measures (51.6% and 49.5%, respectively). The top five categories that were impacted by the COVID-19 pandemic and the policy measures implemented were access to healthcare services and infection prevention supplies (76.6% vs 73.5%), economy (73.7% vs 70.6%), mental health (70.8% vs 67.3%), education (69.4% vs 68.1%), and people’s daily activities (62.6% vs 60.0%) (Table 2).

**Table 2.** Level of impact of COVID-19 from the pandemic and policy measures among participants (*n* = 2,500)

Score	Level	Impact on COVID-19			
		From pandemic		From policy measures	
		*n*	%	*n*	%
**Overall impact**					
≤95	Low	169	6.8	186	7.4
96–143	Moderate	1,040	41.6	1,076	43.0
144–192	High	1,291	51.6	1,238	49.5
Impact from pandemic: Mean = 141.8, SD = 28.1, Median = 145.0, Range = 48–192					
Impact from policy measures: Mean = 139.9, SD = 28.2, Median = 143.0, Range = 48–192					
**Access to healthcare services and infection prevention supplies**					
≤5	Low	153	6.1	171	6.8
6–8	Moderate	432	17.3	492	19.7
9–12	High	1,915	76.6	1,837	73.5
Impact from pandemic: Mean = 9.7, SD = 2.3, Median = 10.0, Range = 3–12					
Impact from policy measures: Mean = 9.5, SD = 2.4, Median = 10.0, Range = 3–12					
**Economy**					
≤15	Low	228	9.1	246	9.8
16–23	Moderate	430	17.2	488	19.5
24–32	High	1,842	73.7	1,766	70.6
Impact from pandemic: Mean = 25.6, SD = 6.1, Median = 27.0, Range = 8–32					
Impact from policy measures: Mean = 25.1, SD = 6.2, Median = 26.0, Range = 8–32					
**Mental health**					
≤9	Low	364	14.5	356	14.2
10–14	Moderate	367	14.7	462	18.5
15–20	High	1,769	70.8	1,682	67.3
Impact from pandemic: Mean = 15.6, SD = 4.8, Median = 17.0, Range = 5–20					
Impact from policy measures: Mean = 15.3, SD = 4.8, Median = 17.0, Range = 5–20					
**Education**					
≤11	Low	164	6.6	182	7.3
12–17	Moderate	601	24.0	616	24.6
18–24	High	1,735	69.4	1,702	68.1
Impact from pandemic: Mean = 19.4, SD = 4.6, Median = 20.0, Range = 6–24					
Impact from policy measures: Mean = 18.8, SD = 4.6, Median = 20.0, Range = 6–24					
**Daily activities**					
≤23	Low	198	7.9	221	8.8
24–35	Moderate	738	29.5	779	31.2
36–48	High	1,564	62.6	1,500	60.0
Impact from pandemic: Mean = 36.9, SD = 8.6, Median = 38.0, Range = 12–48					
Impact from policy measures: Mean = 36.4, SD = 8.7, Median = 38.0, Range = 12–48					
**Cultural/traditional or community ways of life**					
≤9	Low	219	8.8	223	8.9
10–14	Moderate	745	29.8	791	31.6
15–20	High	1,536	61.4	1,486	59.5
Impact from pandemic: Mean = 15.0, SD = 4.1, Median = 15.0, Range = 5–20					
Impact from policy measures: Mean = 14.8, SD = 4.1, Median = 15.0, Range = 5–20					
**Family**					
≤17	Low	1,033	41.3	1,018	40.7
18–26	Moderate	893	35.7	897	35.9
27–36	High	574	23.0	585	23.4
Impact from pandemic: Mean = 19.7, SD = 7.7, Median = 19.0, Range = 9–36					
Impact from policy measures: Mean = 19.8, SD = 7.7, Median = 19.0, Range = 9–36					

### Impact on COVID-19 from the pandemic and policy measures

The top five categories impacted by the COVID-19 pandemic and policy measures were the economy including a decrease in income (65.9% vs 59.6%), insufficient income (65.4% vs 59.0%), temporary furlough from work (62.6% vs 56.1%), loss of income 61.5% vs 55.3%), and layoffs from work (60.4% vs 59.8%). These were followed by the impact on their daily activities including decreased international travel (59.4% vs 54.4%), decreased domestic travel (54.9% vs 50.8%), and decreased shopping at the mall (51.2% vs 47.2%) (Table 3).

**Table 3.** Response to impact of COVID-19 from outbreak and policy measures items among participants (*n* = 2,500)

	Impact of COVID-19 items	Level of impact of COVID-19 from outbreak				Level of impact of COVID-19 on policy measures			
		None*n* (%)	Low*n* (%)	Moderate*n* (%)	High*n* (%)	None*n* (%)	Low*n* (%)	Moderate*n* (%)	High*n* (%)
**Economy**									
1.	Decrease in income	151	210	492	1,647	173	248	588	1,491
		(6.0)	(8.4)	(19.7)	(65.9)	(6.9)	(9.9)	(23.5)	(59.6)
2.	Lack of income	156	217	493	1,634	180	232	613	1,475
		(6.2)	(8.7)	(19.7)	(65.4)	(7.2)	(9.3)	(27.8)	(59.0)
3.	Temporarily furloughed from work	215	206	515	1,564	230	226	642	1,402
		(8.6)	(8.2)	(20.6)	(62.6)	(9.2)	(9.0)	(25.7)	(56.1)
4.	No income	228	215	519	1,538	275	273	558	1,394
		(9.1)	(8.6)	(20.8)	(61.5)	(11.0)	(10.9)	(22.3)	(55.8)
5.	Laid off from work	276	247	468	1,509	244	247	627	1,382
		(11.0)	(9.9)	(18.7)	(60.4)	(9.8)	(9.9)	(25.1)	(55.3)
6.	Increased cost of studying/working from home	163	221	646	1,470	179	264	696	1,361
		(6.5)	(8.8)	(25.8)	(58.8)	(7.2)	(10.6)	(27.8)	(54.4)
7.	Increase income from food delivery	512	441	676	871	515	449	749	787
		(20.5)	(17.6)	(27.0)	(34.8)	(20.6)	(18.0)	(30.0)	(31.5)
8.	Increase income from online sales	626	433	649	792	605	466	702	727
		(25.0)	(17.3)	(26.0)	(31.7)	(24.2)	(18.6)	(28.1)	(29.1)
**Daily activities**									
1.	International travel	391	242	381	1,486	390	271	479	1,360
		(15.6)	(9.7)	(15.2)	(59.4)	(15.6)	(10.8)	(19.2)	(54.4)
2.	Domestic travel	229	299	599	1,373	236	356	639	1,269
		(9.2)	(12.0)	(24.0)	(54.9)	(9.4)	(14.2)	(25.6)	(50.8)
3.	Shopping at the mall	137	337	745	1,281	175	318	826	1,181
		(5.5)	(13.5)	(29.8)	(51.2)	(7.0)	(12.7)	(33.0)	(47.2)
4.	Grocery shopping	106	322	887	1,185	129	349	920	1,102)
		(4.2)	(12.9)	(35.5)	(47.4)	(5.2)	(14.0)	(36.8)	(44.1
5.	Beauty salon	241	385	706	1,168	254	383	793	1,070
		(9.6)	(15.4)	(28.2)	(46.7)	(10.2)	(15.3)	(31.7)	(42.8)
6.	Dining out	158	364	835	1,143	171	374	899	1,056
		(6.3)	(15.5)	(33.4)	(45.7)	(6.8)	(15.0)	(36.0)	(42.2)
7.	Attending parties	265	439	699	1,097	268	455	791	986
		(10.6)	(17.6)	(28.0)	(43.9)	(10.7)	(18.2)	(31.6)	(39.4)
8.	Outdoor exercise	185	388	910	1,017	205	476	859	960
		(7.4)	(15.5)	(36.4)	(40.7)	(8.2)	(19.0)	(34.4)	(38.4)
9.	Banking services	205	458	825	1,012	199	391	964	946
		(8.2)	(18.3)	(33.0)	(40.5)	(8.0)	(17.3)	(38.6)	(37.8)
10.	Entertainment	384	473	674	969	362	464	778	896
		(15.4)	(18.9)	(27.0)	(38.8)	(14.5)	(18.6)	(31.1)	(35.8)
11.	Meeting with close friends	266	545	871	818	276	581	885	758
		(10.6)	(21.8)	(34.8)	(32.7)	(11.0)	(23.2)	(35.4)	(30.3)
12.	Meeting with romantic partners	505	496	758	741	468	554	754	724
		(20.2)	(19.8)	(30.3)	(29.6)	(18.7)	(22.2)	(30.2)	(29.0)
**Mental health**									
1.	Anxiety about layoffs	309	256	544	1,391	304	305	625	1,266
		(12.4)	(10.2)	(21.8)	(55.6)	(12.2)	(12.2)	(25.0)	(50.6)
2.	Stress from unemployment	306	290	524	1,380	325	331	623	1,221
		(12.2)	(11.6)	(21.0)	(55.2)	(13.0)	(13.2)	(24.9)	(48.8)
3.	Anxiety about finding a job	282	273	622	1,323	317	322	653	1,208
		(11.3)	(10.9)	(24.9)	(52.9)	(12.7)	(12.9)	(26.1)	(48.3)
4.	Stress from being temporarily furloughed from work	323	275	609	1,293	295	327	707	1,171
		(12.9)	(11.0)	(24.4)	(51.7)	(11.8)	(13.1)	(28.3)	(46.8)
5.	Depression from being alone	479	354	646	1,021	464	419	654	963
		(19.2)	(14.2)	(25.8)	(40.8)	(18.6)	(16.8)	(26.2)	(38.5)
**Education**									
1.	Students delayed graduation	155	322	676	1,347	165	285	791	1,259
		(6.2)	(12.9)	(27.0)	(53.9)	(6.6)	(11.4)	(31.6)	(50.4)
2.	Students cannot go to tutoring	151	324	734	1,291	159	328	827	1,186
		(6.0)	(13.0)	(29.4)	(51.6)	(6.4)	(13.1)	(33.1)	(47.4)
3.	Students do not have access to devices and accessories for online learning	219	349	783	1,149	237	380	785	1,098
		(8.8)	(14.0)	(31.3)	(46.0)	(9.5)	(15.2)	(31.4)	(43.9)
4.	Students do not have support for online learning	206	390	766	1,138	211	398	814	1,077
		(8.2)	(15.6)	(30.6)	(45.5)	(8.4)	(15.9)	(32.6)	(43.1)
5.	Students do not have access to Internet for online learning	243	386	750	1,121	217	367	852	1,064
		(9.7)	(15.4)	(30.0)	(44.8)	(8.7)	(14.7)	(34.1)	(42.6)
6.	Students do not have skills for online learning	198	403	819	1,080	206	400	859	1,035
		(7.9)	(16.1)	(32.8)	(43.2)	(8.2)	(16.0)	(34.4)	(41.4)
**Access to healthcare services and infection prevention supplies**									
1.	Lack of mask	165	298	787	1,250	179	345	803	1,173
		(6.6)	(11.9)	(31.5)	(50.0)	(7.2)	(13.8)	(32.1)	(46.9)
2.	Difficulty in accessing healthcare	141	314	815	1,230	158	331	880	1,131
		(5.6)	(12.6)	(32.6)	(49.2)	(6.3)	(13.2)	(35.2)	(45.2)
3.	Lack of alcohol hand gel	174	333	820	1,173	191	356	840	1,113
		(7.0)	(13.3)	(32.8)	(46.9)	(7.6)	(14.2)	(33.6)	(44.5)
**Cultural/traditional or community ways of life**									
1.	Decrease in support for each other in community activities	189	469	788	1,054	186	495	859	960
		(7.6)	(18.8)	(31.5)	(42.2)	(7.4)	(19.8)	(34.4)	(38.4)
2.	Decrease in religious activities	235	500	760	1,005	227	548	785	940
		(9.4)	(20.0)	(30.4)	(40.2)	(9.1)	(21.9)	(31.4)	(37.6)
3.	Increase screening and surveillance PIQ from other areas	249	496	785	970	264	525	802	909
		(10.0)	(19.8)	(31.4)	(38.8)	(10.6)	(21.0)	(32.1)	(36.4)
4.	Decrease preserved traditions and culture	189	543	812	956	186	568	849	897
		(7.6)	(21.7)	(32.5)	(38.2)	(7.4)	(22.7)	(34.0)	(35.9)
5.	Increase screening and surveillance of PIQ living in community	237	502	910	851	243	521	919	817
		(9.5)	(20.1)	(36.4)	(34.0)	(9.7)	(20.8)	(36.8)	(32.7)
**Family**									
1.	Lack of caregiver for bedridden	771	500	635	594	768	529	670	533
		(30.8)	(20.0)	(25.4)	(23.8)	(30.7)	(21.2)	(26.8)	(21.3)
2.	Lack of caregivers for older adults	731	521	682	566	723	575	672	530
		(29.2)	(20.8)	(27.3)	(22.6)	(28.9)	(23.0)	(26.9)	(21.2)
3.	Lack of caregivers for children	760	578	641	521	727	596	677	500
		(30.4)	(23.1)	(25.6)	(20.8)	(29.1)	(23.8)	(27.1)	(20.0)
4.	Decrease in community relationships	569	716	709	506	584	711	726	479
		(22.8)	(28.6)	(28.4)	(20.2)	(23.4)	(28.4)	(29.0)	(19.2)
5.	Decrease in sexual activity and pregnancy among teenagers	936	653	522	389	932	649	560	359
		(37.4)	(26.1)	(20.9)	(15.6)	(37.3)	(26.0)	(22.4)	(14.4)
6.	Decrease in family relationships	924	642	593	341	890	620	662	328
		(37.0)	(25.7)	(23.7)	(13.6)	(35.6)	(24.8)	(26.5)	(13.1)
7.	Increase fear of having sexual activity with husband, wife, or romantic partner	1,046	610	508	336	1,015	630	538	317
		(41.8)	(24.4)	(20.3)	(13.4)	(40.6)	(25.2)	(21.5)	(12.7)
8.	Increase arguing with romantic partner	1,189	593	441	277	1,129	613	492	266
		(47.6)	(23.7)	(17.6)	(11.1)	(45.2)	(24.5)	(19.7)	(10.6)
9.	Increase broken family or divorce	1,312	541	388	259	1,233	574	449	244
		(52.5)	(21.6)	(15.5)	(10.4)	(49.3)	(23.0)	(18.0)	(9.8)

### Level of anxiety, stress, and depression from the COVID-19 pandemic

Some participants reported extremely severe anxiety (11.0%), extremely severe stress (4.2%), and extremely severe depression (6.3%) from the COVID-19 outbreak (Table 4).

**Table 4.** Level of anxiety, stress, and depression from the COVID-19 pandemic among participants (*n* = 2,500)

Level	Psychological impact					
	Anxiety		Stress		Depression	
	*n*	%	*n*	%	*n*	%
Normal	1,688	67.5	1,898	75.9	1,791	71.6
Mild	261	10.4	172	6.9	227	9.1
Moderate	180	7.2	194	7.8	216	8.6
Severe	95	3.8	132	5.3	108	4.3
Extremely severe	276	11.0	104	4.2	158	6.3

### Comparing effects of the COVID-19 pandemic to policy measures

The weighted effect of the impact of COVID-19 was statistically (*p* < .001) greater than that of governmental policy measures on family (0.664 vs 0.618), education (0.562 vs 0.557), and economy (0.643 vs 0.572). The weighted effect of the impact from governmental policy measures was statistically (*p* < .001) greater than that of COVID-19 on daily activities (0.675 vs 0.651), cultural/traditional or community ways of life (0.769 vs 0.736), access to healthcare services and infection prevention supplies (0.410 vs 0.390), and mental health (0.625 vs 0.584) (Table 5).

**Table 5.** Comparison of the weighted effect of impact of the COVID-19 pandemic and policy measures among participants

Impact category	Weighted effect of impact from COVID-19 pandemic and policy measures*					
	Overall(*n* = 2,500)	Bangkok(*n* = 500)	Chonburi(*n* = 500)	Chiang Mai(*n* = 500)	Nakhon Ratchasima(*n* = 500)	Yala(*n* = 500)
Daily activities	0.651	0.602	0.561	0.758	0.736	0.749
	(0.675)	(0.594)	(0.604)	(0.748)	(0.692)	(0.748)
Family	0.664	0.619	0.708	0.687	0.649	0.551
	(0.618)	(0.612)	(0.656)	(0.619)	(0.638)	(0.568)
Cultural/traditional or community ways of life	0.736	0.634	0.703	0.697	0.702	0.699
	(0.769)	(0.729)	(0.822)	(0.693)	(0.749)	(0.788)
Education	0.562	0.757	0.657	0.554	0.495	0.569
	(0.557)	(0.638)	(0.678)	(0.563)	(0.552)	(0.549)
Access to healthcare services and infection prevention supplies	0.390	0.534	0.447	0.352	0.317	0.462
	(0.410)	(0.465)	(0.413)	(0.491)	(0.286)	(0.432)
Economy	0.643	0.696	0.727	0.556	0.667	0.592
	(0.572)	(0.640)	(0.631)	(0.521)	(0.654)	(0.554)
Mental health	0.584	0.658	0.674	0.540	0.509	0.508
	(0.625)	(0.611)	(0.692)	(0.572)	(0.627)	(0.518)

* Weighted effect of impact from policy measures was presented in parenthesis.

## Discussion

This study achieved a 100% response rate. This may be because this study attributes its success to a clear and concise questionnaire, pre-testing for construct and face validity, and effective communication by researchers emphasizing the survey’s benefits, confidentiality, and anonymity. Accessibility was ensured by 7 researchers and 125 health volunteers visiting participants’ homes, allowing for immediate completion or within 2 weeks at the participants’ convenience. Incentives of 100 baht (approximately three dollars) for participants were offered.

This study found that about half of the participants felt the effects of COVID-19 and subsequent governmental policy measures affected them at the highest level. The weighted effects of the impact of the COVID-19 pandemic and policy measures were different.

More than 60.0% of participants felt the economic impact of the COVID-19 pandemic and the policy measures were the most profound, including a decrease in income, being furloughed from work, or being unemployed. The findings are consistent with global reports^10,11^ and studies from the US^12^ and Thailand.^11,13,22^

The impact from the COVID-19 pandemic and policy measures had significant effects on people’s daily activities including international travel, domestic travel, shopping at the mall, grocery shopping, visits to beauty salons, dining out, attending parties, outdoor exercise, visiting banks, entertainment, meeting close friends, and meeting romantic partners. These findings are congruent with systematic reviews^1,2^ and studies from Turkey^23^ and the US.^24^

Some participants in this study also had psychological problems including anxiety about layoffs, stress from unemployment, anxiety about finding a job, stress from being temporarily furloughed from work, and depression from being alone. This is similar to findings from studies conducted in the US,^24^ Spain,^25^ Italy,^26^ and Thailand.^8,16,27–29^

This study also found that some participants had problems related to their education including delays in their graduation, not being able to attend tutoring, not having access to devices and accessories for online learning, lack of support for online learning, lack of Internet access for online learning, and lack of skills for online learning. This is congruent to findings from studies in Spain,^4^ Saudi Arabia,^30^ and Thailand.^8^

Participants in this study also reported problems with access to healthcare services, which was similar to studies from Chile,^31^ Nigeria,^32^ and Thailand.^8^

In addition, this study found that participants had difficulty accessing infection prevention supplies due to a shortage of masks^33,34^ and alcohol-based hand sanitizers.^34^

Some participants in this study had issues with cultural/traditional or community ways of life including decreased support for each other in community activities, decreased religious activities, increased screening and surveillance of people in quarantine (PIQ) from other areas, inability to practice traditions and culture, and increased screening and surveillance of PIQ living in the community. This is on par with studies from the WHO,^7^ France,^3,6^ Ghana,^35^ and Thailand.^8^

Participants in this study also experienced familial problems including lack of caregivers for bedridden family members, older adults, and children; a decrease in community relationships; a decrease in sexual activity and pregnancy among teenagers; a decrease in family relationships; fear of engaging in sexual activities with a spouse or romantic partner; arguments with a romantic partner; and having a broken family or divorce. These findings are congruent with one systematic review^30^ and another study done in Thailand.^8^

As determined by this study, the overall weighted effects of the impact of COVID-19 and policy measures were statistically different. The strength of this effect varies among different categories. In the case of being affected by the COVID-19 pandemic, people in urban areas, such as Bangkok and Chonburi, were affected by changes in education more than other categories, which further affected the economy. This is due to the fact that parents have to take time off work to take care of their children attending school online at home. On the other hand, people in rural areas such as Chiang Mai, Nakhon Ratchasima, and Yala found that their cultural/traditional or community ways of life and daily activities were impacted more than other categories. The policy measures affected the economy and mental health as reported by people in urban areas, including Bangkok and Chonburi. People in rural areas, including Chiang Mai, Nakhon Ratchasima, and Yala, reported that daily activities remained an important aspect of their lives. People in Nakhon Ratchasima and Yala reported that cultural/traditional or community ways of life were of higher importance than other categories, which was different from Chiang Mai where access to healthcare services and infection prevention supplies were most important.

The impact on cultural/traditional or community ways of life and daily activities led to reduced interactions within the community and changed the social norms of people who lived in rural areas and usually had close relationships. The economic and psychological impacts result in a reduced quality of life and, in some cases, may lead to suicide.

This study’s findings can be used to develop policies or other methods to reduce the impact of future pandemics on the population. They can also be used as a guide when looking at the severity of aspects of the impact of COVID-19 and policy measures, especially in urban and rural areas where impacts are different depending on peoples’ lifestyles, cultural/traditional or community ways of life, and social norms. Policymakers should develop strategies to provide appropriate support based on how the categories are affected in specific areas (rural vs. urban). If the dominant impact is caused by the COVID-19 pandemic, the government should focus on reducing the spread of COVID-19 and decreasing the incidence of new cases. If the dominant impact is caused by policy measures, especially the country lockdown by which people were hit hardest, the government should focus on changing policies as appropriate based on the prevalence of COVID-19 and ensuring the balance of the 7 categories, especially the economy, daily activities, and cultural/traditional or community ways of life. Information from this study can be used to devise policies in accordance with the dynamics of the COVID-19 pandemic. The government should change policies and adopt measures appropriately to the pandemic situation and balance these measures with other goals such as the economy, education, and way of life. The government should adopt disease control measures based on risk areas instead of implementing a comprehensive lockdown of the entire country.

Based on our literature review and to the best of our knowledge, this is the first study that has looked at the impact of the COVID-19 pandemic and compared the impact of the pandemic and government policy measures. The impacts of the COVID-19 pandemic and policy measures are interconnected, as the latter are responses to the challenges posed by the pandemic, yet they also have distinct characteristics and implications. Recognizing the dynamic of the pandemic and policy effectiveness is crucial, as it makes identifying and comparing their actual effects challenging. Researchers addressed this complexity by explaining impact definitions to participants before questionnaire completion, allowing time for questions and answers.

The results of this study are strengthened by it being a multi-site study with a large sample size and geographic and demographic variability in Thailand. It is important to recognize that individuals within these regions may not be representative of all Thai citizens. The limitation, however, is that it used a self-reported questionnaire which may lead to information bias. This was mitigated by the high content validity of the questionnaire as well as informing all participants about the importance of accuracy when responding to the questions.

## Conclusion

About half of the participants in this study felt that the impacts of COVID-19 and the resulting governmental policy measures affected their lives. However, the weighted effect of the impact of the COVID-19 pandemic and the policy measures affected areas and categories differently. More than 60% of participants felt that the economic impact of COVID-19 and the policy measures were the most profound, leading to a decrease in income, lack of income, being temporarily furloughed from work, or being unemployed. Based on this study, we recommend that the government should change policies and adopt measures appropriately to the pandemic situation to ensure that there is a balance with other goals. The government should provide widespread support to reduce the impacts of the pandemic and people’s suffering.

## References

[1] El Keshky MES , Basyouni SS , Al Sabban AM. Getting through COVID-19: the pandemic’s impact on the psychology of sustainability, quality of life, and the global economy – a systematic review [published correction appears in *Front Psychol*. 2021 May 26;12:700815]. Front Psychol 2020;11:585897. 33281683 10.3389/fpsyg.2020.585897 PMC7688781

[2] Haleem A , Javaid M , Vaishya R. Effects of COVID-19 pandemic in daily life. Curr Med Res Pract 2020;10:78–79. 32292804 10.1016/j.cmrp.2020.03.011 PMC7147210

[3] Raude J. Determinants of preventive behaviors in response to the COVID-19 pandemic in France: comparing the sociocultural, psychosocial and social cognitive explanations. 10.31234/osf.io/4yvk2. Accessed June 14, 2020. PMC7734102 33329241

[4] Sintema EJ. Effect of COVID-19 on the performance of grade 12 students: implications for STEM education. EURASIA J Math Sci Tech Ed 2020;16:em1851.

[5] Moynihan R , Sanders S , Michaleff ZA , et al. Impact of COVID-19 pandemic on utilisation of healthcare services: a systematic review. BMJ Open 2021;11:e045343. 10.1136/bmjopen-2020-045343 PMC7969768 33727273

[6] Raude J. Determinants of preventive behaviors in response to the COVID-19 pandemic in France: comparing the sociocultural, psychosocial and social cognitive explanations. 10.31234/osf.io/4yvk2. Accessed June 14, 2020. PMC7734102 33329241

[7] World Health Organization [WHO]. Social stigma associated with COVID-19. 2020. https://www.who.int/docs/default-source/searo/thailand/covid19-stigma-guide-th-final.pdf?sfvrsn=1eebbcac_0. Accessed June 17, 2020.

[8] Kasatpibal N. Knowledge, Attitude, and Preventative Practices Taken by the Thai Population Regarding People with COVID-19 and People in Quarantine. Chiang Mai: Faculty of Nursing, Chiang Mai University; 2020.

[9] Sintema EJ. Effect of COVID-19 on the performance of grade 12 students: implications for STEM education. EURASIA J Math Sci Tech Ed 2020;16:em1851.

[10] United Nations Conference on Trade and Development. Global trade impact of coronavirus (COVID-19) epidemic. 2020. https://unctad.org/en/PublicationsLibrary/ditcinf2020d1.pdf. Accessed June 14, 2020.

[11] Sumner A , Hoy C , Ortiz-Juarez E. Estimates of the impact of COVID-19 on global poverty. WIDER Working Paper 2020/43. 2020; Helsinki: UNU-WIDER. 10.35188/UNU-WIDER/2020/800-9.

[12] Parker K , Horowitz JM , Brown A. About Half of Lower-income Americans Report Household Job or Wage Loss Due to COVID-19. Social & Demographic Trends Project, Pew Research Center; 2020. https://www.pewresearch.org/social-trends/wp-content/uploads/sites/3/2020/04/PSDT_04.21.20_covidfinance_FULL.REPORT.pdf

[13] Satayanurug A , Visetpricha B , Pintobtang P , et al. The urban slum people in a changing society. https://www.isranews.org/article/isranews/download/18017/87576/18.html. Accessed June 12, 2020.

[14] Ho CS , Chee CY , Ho RC. Mental health strategies to combat the psychological impact of COVID-19 beyond paranoia and panic. Ann Acad Med Singapore 2020;49:1–3. 32200399

[15] Hossain MM , Sultana A , Purohit N. Mental health outcomes of quarantine and isolation for infection prevention: a systematic umbrella review of the global evidence. Epidemiol Health 2020;42:e2020038. 32512661 10.4178/epih.e2020038 PMC7644933

[16] Apisarnthanarak A , Siripraparat C , Apisarnthanarak P , et al. Patients’ anxiety, fear, and panic related to coronavirus disease 2019 (COVID-19) and confidence in hospital infection control policy in outpatient departments: a survey from four Thai hospitals. Infect Control Hosp Epidemiol 2021;42:1288–1290. 33023718 10.1017/ice.2020.1240 PMC7573456

[17] Zhang Y , Ma ZF. Impact of the COVID-19 pandemic on mental health and quality of life among local residents in Liaoning Province, China: a cross-sectional study. Int J Environ Res Public Health 2020;17:2381. 32244498 10.3390/ijerph17072381 PMC7177660

[18] Choi EP , Hui BP , Wan EY. Depression and anxiety in Hong Kong during COVID-19. Int J Environ Res Public Health 2020;17:3740. 32466251 10.3390/ijerph17103740 PMC7277420

[19] Torales J , O’Higgins M , Castaldelli-Maia JM , Ventriglio A. The outbreak of COVID-19 coronavirus and its impact on global mental health. Int J Soc Psychiatry 2020;66:317–320. 32233719 10.1177/0020764020915212

[20] Gao J , Zheng P , Jia Y , et al. Mental health problems and social media exposure during COVID-19 outbreak. Plos One 2020;15:e0231924. 32298385 10.1371/journal.pone.0231924 PMC7162477

[21] Oei TP , Sawang S , Goh YW , Mukhtar F. Using the depression anxiety stress scale 21 (DASS-21) across cultures. Int J Psychol 2013;48:1018–1029. 23425257 10.1080/00207594.2012.755535

[22] International Labour Organization. COVID-19: impact on migrant workers and country response in Thailand. 2020. https://www.ilo.org/wcmsp5/groups/public/---asia/---ro-bangkok/---sro-bangkok/documents/briefingnote/wcms_741920.pdf. Accessed May 15, 2021.

[23] Ali H , Yilmaz G , Fareed Z , Shahzad F , Ahmad M. Impact of novel coronavirus (COVID-19) on daily routines and air environment: evidence from Turkey. Air Qual Atmos Health 2021;14(3):381–387. 10.1007/s11869-020-00943-2 32983281 PMC7508423

[24] Giuntella O , Hyde K , Saccardo S , Sadoff S. Lifestyle and mental health disruptions during COVID-19. Proc Natl Acad Sci USA 2021;118:e2016632118. 33571107 10.1073/pnas.2016632118 PMC7936339

[25] Rodríguez-Rey R , Garrido-Hernansaiz H , and Collado S. Psychological impact and associated factors during the initial stage of the coronavirus (COVID-19) pandemic among the general population in Spain. Front Psychol 2020;11:1540. 32655463 10.3389/fpsyg.2020.01540 PMC7325630

[26] Saladino V , Algeri D , Auriemma V. The psychological and social impact of COVID-19: new perspectives of well-being. Front Psychol 2020;11:577684. 33132986 10.3389/fpsyg.2020.577684 PMC7561673

[27] Thatrimontrichai A , Weber DJ , Apisarnthanarak A. Mental health among healthcare personnel during COVID-19 in Asia: a systematic review. J Formos Med Assoc 2021;120:1296–1304. 33581962 10.1016/j.jfma.2021.01.023

[28] Narupaves N , Kulworasreth P , Manaanuntakul N , Warren DK , Weber DJ , Apisarnthanarak A. Coronavirus disease 2019 (COVID-19) preparedness in a Thai international school: emotional health and infection control practices [published correction appears in *Infect Control Hosp Epidemiol*. 2022;43(9):1311]. Infect Control Hosp Epidemiol 2022;43:1307–1309. 34016203 10.1017/ice.2021.236 PMC8193194

[29] Apisarnthanarak A , Apisarnthanarak P , Siripraparat C , Saengaram P , Leeprechanon N , Weber DJ. Impact of anxiety and fear for COVID-19 toward infection control practices among Thai healthcare workers. Infect Control Hosp Epidemiol 2020;41:1093–1094. 32507115 10.1017/ice.2020.280 PMC7298087

[30] Alghamdi AA. Impact of the COVID-19 pandemic on the social and educational aspects of Saudi university students’ lives. Plos One 2021;16:e0250026. 33852627 10.1371/journal.pone.0250026 PMC8046245

[31] Núñez A , Sreeganga SD , Ramaprasad A. Access to healthcare during COVID-19. Int J Environ Res Public Health 2021;18:2980. 33799417 10.3390/ijerph18062980 PMC7999346

[32] Okereke M , Ukor NA , Adebisi YA , et al. Impact of COVID-19 on access to healthcare in low- and middle-income countries: current evidence and future recommendations. Int J Health Plann Manage 2021;36:13–17. 32857892 10.1002/hpm.3067

[33] Ji D , Fan L , Li X , Ramakrishna S. Addressing the worldwide shortages of face masks. BMC Mater 2020;2:9. 32835173 10.1186/s42833-020-00015-w PMC7393630

[34] Kampf G , Scheithauer S , Lemmen S , Saliou P , Suchomel M. COVID-19-associated shortage of alcohol-based hand rubs, face masks, medical gloves, and gowns: proposal for a risk-adapted approach to ensure patient and healthcare worker safety. J Hosp Infect 2020;105:424–427. 32360355 10.1016/j.jhin.2020.04.041 PMC7190502

[35] Osei-Tutu A , Affram AA , Mensah-Sarbah C , Dzokoto VA , Adams G. The impact of COVID-19 and religious restrictions on the well-being of Ghanaian Christians: the perspectives of religious leaders. J Relig Health 2021;60:2232–2249. 34014473 10.1007/s10943-021-01285-8 PMC8136111

